# Theoretical analysis of OLED performances of some aromatic nitrogen-containing ligands

**DOI:** 10.55730/1300-0527.3571

**Published:** 2023-05-24

**Authors:** Mustafa ELİK

**Affiliations:** Department of Mathematics and Science Education, Faculty of Education, Sivas Cumhuriyet University, Sivas, Turkiye

**Keywords:** Cyclic structures including nitrogen atom, charge transfer, DFT, OLED

## Abstract

It is well-known that tris(8-hydroxyquinoline) aluminum (Alq3) complex and N,N′diphenyl-N,N′-bis(3-methylphenyl)-1,1′-diphenyl-4,4′-diamine compound (TPD) are widely used as electron transfer material (ETL) and hole transfer material (HTL) in organic light emitting diode (OLED) structure, respectively. Considering the reference materials, in the present work, the OLED performances of some cyclic aromatic structures such as 4,4′azopyridine [AZPY], 4,4′-bipyridine [BIPY], 1,2-bis[4′-(4-methylphenyl)2,2′:6′2″-terpyridin6-yl]ethyne (BISTERPY), 5,5′-diamino-2,2′-bipyridine (DABP), dipyrido[3,2-a:2′,3′c]phenazine (DPP), 4,7-phenanthroline (PHEN) including nitrogen atom have been theoretically analyzed. It is important to note that B3LYP/6-31G(d) and B3LYP/TZP levels of the theory were taken into account for the calculations about monomeric and dimeric structures, respectively. Additionally, the calculations of the mentioned monomeric form were performed at B3LYP-D3/6-31G, CAM-B3LYP/6-31G and ωB97X-D/6-31G(d) levels. For a detailed theoretical analysis, the reorganization energies (λe and λh), adiabatic and vertical ionization potentials and electron affinities, the effective transfer integrals (Ve and Vh), and the charge transfer rates (We and Wh) of all compounds were computed by means of computational chemistry tools. In the light of calculated parameters, it is determined that these mentioned aromatic cyclic structures will be used in which layers of OLED structure. The results obtained in this study will be helpful in the design and applications of new molecules as OLED materials in the future.

## 1. Introduction

Nowadays, organic light emitting diodes having many important applications in the science are among the subjects of which chemists and physicists are interested in. It should be noted that in the past 30 years, the scientific literature witnessed the important developments about organic light emitting diodes. These diodes are widely used in screens of many electronic devices. In the studies about OLEDs, there are significant attempts to improve the effectiveness of these materials. For this aim, many organic molecules and metal complexes have been proposed as effective and useful OLED materials. In this paper, in the light of important developments about OLEDS, we will investigate the OLED properties of some organic compounds reported in the literature with the help of suitable theoretical analysis and parameters.

One of the principal goals of theoretical chemists is to develop new and useful rules and theories to explain the reactivity properties and to predict the directions and mechanisms of chemical reactions [1–10]. Density functional theory (DFT) that was revealed in accordance with this goal uses the electron density to identify the reactivity or stability [11–17]. It should be noted that hybrid functional such as B3LYP and M062 are widely preferred in the studies regarding the explanation of reactivity properties of molecules [18–24]. Additionally, in the studies also researching organic light emitting diode performances of various molecules, the aforementioned functional have been used [25–31]. In an edited book entitled “Conceptual Density Functional Theory and Its Applications in the Chemical Domain”, İslam and Kaya provided detailed information about the applications and achievements of hybrid functional [32].

The aim of this article is to investigate the relation with molecular structure of OLED behaviors of some aromatic cyclic structures including nitrogen atom which are shown in Scheme. Within the framework of obtained data, the best OLED material among studied compounds will be determined. Thanks to the results obtained theoretical data here, we will propose the using of the mentioned molecules in the design of new OLED materials as experimental. It is important to note that this study is the first attempt investigating the OLED properties of the compounds considered.

## 2. Methods

It is important to note that all computations related to monomeric forms of studied aromatic cycles including nitrogen atom were performed using Gaussian 16 [33] and GaussView 6 [34] package programs. In the calculations about dimeric forms of the molecules, ADF2019 program [35, 36] was preferred because charge transfer integrals can be easily obtained using this program. It was shown in optimization processes that the imaginer frequencies of all molecules considered in the study correspond to local minima.

It is apparent from the literature that in such studies hybrid B3LYP functional are used to analyze the charge transport properties of various molecular groups [37–42]. In the study, charge transfer properties and chemical behaviors of the molecules considered were recorded in the light of same hybrid functional. For monomer and dimer calculations, the B3LYP/631G(d) and B3LYP/TZP levels were used, respectively. The calculations of the mentioned monomeric structures were also performed at B3LYP-D3/6-31G, CAM-B3LYP/6-31G and ωB97X-D/6-31G(d) levels.

To compute the electronic hopping rate, K, Marcus and Hush [43–46] introduced the following equation, which has been extensively preferred in previous similar studies [47,48].


(1) $$W_{e/h}=\frac{{V_{e/h}}^{2}}{\hbar }{\left(\frac{\pi }{\lambda_{e/h}k_{B}T}\right)}^{1/2}\text{exp }\left(-\frac{\lambda_{e/h}}{4k_{B}T}\right),$$

where V is the effective (generalized) transfer integral which represents the electronic coupling between two adjacent molecules. λ denotes the reorganization energies, k_B_ is the

Boltzmann constant, ћ and T stand for Dirac constant and absolute temperature which was taken as 298.15 K in our calculations, respectively.

In the equations given below, *ϕ*_1_*^H^*^/^*^L^* and *ϕ*_2_*^H^*^/^*^L^* represent the localized molecular orbitals of neighboring monomers. h stands for Kohn-Sham Hamiltonian of a dimeric structure and J, S, and E are charge transfer integral, overlap matrix and site energy of monomer, respectively.


(2) $$\begin{array}{l} J_{12}=\langle {\phi_{1}}^{H/L}|h_{KS}|{\phi_{2}}^{H/L}\rangle \\ S_{12}=\langle {\phi_{1}}^{H/L}|{\phi_{2}}^{H/L}\rangle \\ E_{1}=\langle {\phi_{1}}^{H/L}|h_{KS}|{\phi_{1}}^{H/L}\rangle \\ E_{2}=\langle {\phi_{2}}^{H/L}|h_{KS}|{\phi_{2}}^{H/L}\rangle \end{array}$$

After the parameters appearing in Eq. 2 are calculated, the electronic coupling (V) can be calculated with the help of Eq. 3. Instead of dealing with such complex calculations, considering energy splitting procedure and using ADF program, electronic coupling can be directly calculated.


(3) $$V_{e/h}=\frac{J_{12}-S_{12}(E_{1}+E_{2})/2}{1-{S_{12}}^{2}}$$

Reorganization energy (λ) can be divided into two classes as external reorganization energy (λ_ext_) and internal reorganization energy (λ_int_). In similar studies, λ_ext_ values have been ignored because it directs electronic energy change and nuclear polarization in biological environments and have small values for condensed-state systems. Therefore, in the analysis of OLED performances of studied compounds, we focused on only λ_int_ and determined electron reorganization energy (λ_e_) and hole reorganization energy (λ_h_) by means of the following equations.


(4) $$\lambda_{e}=(E_{0}^{-}-E_{-}^{-})+(E_{-}^{0}-E_{0}^{0})$$

(5) $$\lambda_{h}=(E_{0}^{+}-E_{+}^{+})+(E_{+}^{0}-E_{0}^{0})$$

To obtain the adiabatic ionization energy (IP) and adiabatic electron affinity (EA) of studied compounds, Equations 6 and 7 were used.


(6) $$IPa=E_{+}^{+}-E_{0}^{0}$$

(7) $$EAa=E_{0}^{0}-E_{-}^{-}$$

In the equations given above, $E_{0}^{-}\;(E_{0}^{+})$ represents energy of the anion (cation) calculated considering the structure of the neutral form of the studied molecules. As a similar manner, $E_{-}^{-}\;(E_{+}^{+})$ denotes the energy of the anionic (cationic) form determined with the optimized anionic (cationic) structure, $E_{-}^{0}\;(E_{+}^{0}.)$ stands for the energy of the neutral molecule calculated at the anion (cationic) form. Lastly, E_0_^0^ indicates the energy of the neutral form of molecule at the ground state.

Conceptual density functional theory (CDFT) presents the chemical potential (μ) as the first derivative with respect to number of electrons (N) of total electronic energy (E) at a constant external potential, υ(r). Electronegativity (χ) is given as the negative of the chemical potential. Kaya and Kaya defined the chemical hardness (η) as the resistance towards electron cloud polarization or deformation of chemical compounds. In the CDFT, absolute hardness is given as second derivative of electronic energy (E) with respect to the number of electrons (N) [49].


(8) $$\mu =-\chi ={\left[\frac{\partial E}{\partial N}\right]}_{\nu (r)}$$

(9) $$\eta =\frac{1}{2}{\left[\frac{\partial^{2}E}{\partial N^{2}}\right]}_{\nu (r)}$$

Using the finite difference approach, Pearson and Parr presented the mathematical relation with ground state ionization energy (I_gs_) and electron affinities (A_gs_) of chemical species of electronegativity. The mentioned mathematical relations are given as [50]:


(10) $$\chi =\frac{I_{gs}+A_{gs}}{2},$$

(11) $$\eta =\frac{I_{gs}-A_{gs}}{2}.$$

Another important parameter of the chemical reactivity studies is electrophilicity index (ω) introduced by Parr et al. This index is mathematically calculated as [51]:


(12) $$\omega =\chi^{2}/2\eta .$$

In 2007, Gázquez et al. proposed two new parameters known as electrodonating power (ω^−^) and electroaccepting power (ω^+^) to analyze the electron donating and electron accepting capabilities of the molecular systems. The derived equations for the calculations of these parameters are given as [52]:


(13) $$\omega^{+}={\left(I_{gs}+3A_{gs}\right)}^{2}/(16(I_{gs}-A_{gs})),$$

(14) $$\omega^{-}={\left(3I_{gs}+A_{gs}\right)}^{2}/(16(I_{gs}-A_{gs})).$$

## 3. Results and discussion

### 3.1. Reorganization energies

It is well-known that reorganization energies are widely considered in the prediction of the performances of organic light emitting diodes. Many researchers have focused on reorganization energy parameters to analyze the charge transfer ratios of various organic molecule groups. In the present work, the reference materials are tris(8-hydroxyquinoline) aluminum complex (Alq3) which is an electron transfer material (ETL) and N, N′-diphenyl-N,N′-bis(3-methylphenyl)-1,1′-diphenyl-4,4′-diamine (TPD) which is an hole transfer material (HTL) [53–58]. Here, it should be noted that the electron reorganization energy of Alq3 has been reported as 0.276 eV, on the other hand, the hole reorganization energy of TPD is 0.290 Ev [59,60]. The aim of scientists studying OLED performances of the compounds is to propose new and more useful materials compared to these reference materials. Calculated reorganization energies of studied organic aromatic compounds are presented in Table 1 in detail. Calculations made using different computational levels are in good agreement. The trends of all calculated parameters are almost the same. It is apparent from the data given in the related table, BISTERPY can be used as both electron transfer material and hole transfer material because the lower reorganization energy value represents more charge transfer ratio as seen from Marcus-Hush equation given by Eq. 1. This compound is an ambipolar charge transfer material because a molecule having both ETL and HTL material property can be also used as ambipolar charge transfer material. In addition to this information, it can be said that DPP compound is a good candidate to use as an ETL material. The compounds having high electron reorganization energy values such as DABP can be preferred as electron blocking material (EBL). On the other hand, the compounds having high hole reorganization energy values such as AZPY can be preferred as hole blocking material (HBL).

### 3.2. Ionization potentials, ele ctron affinities and chemical hardness, electrophilicity and polarizability values

Ionization energy and electron affinity are parameters that provide important clues about electron donating and electron accepting powers of chemical species. In the conceptual density functional theory, many reactivity descriptors like hardness, softness, chemical potential and electronegativity have been associated with ionization energy and electron affinity values of molecules. Koopmans’ theorem [61] presents an alternative approach to predict the ionization energy and electron affinity values of molecules. According to this theorem, ionization energy and electron affinity values of any chemical species correspond to the negative of its HOMO and LUMO orbital energies, respectively. It is important to note that the chemical hardness values obtained by means of adiabatic ionization energy and adiabatic electron affinity are closer to experimental counterparts compared to determined chemical hardness values using Koopmans’ theorem. For that reason, in the determining of chemical hardness values of our aromatic structures, we used their adiabatic ionization energy and electron affinity values calculated using computational tools. It is well-known from the literature that smaller IP and larger EA values correspond to better hole and electron transport, respectively. It is seen from Table 2 that DABP has the lowest IP values among studied compounds. Therefore, the mentioned compound can be used as a hole injection material. On the other hand, one can say that AZPY molecule with the highest EA value is a good electron injection material. Within the framework of maximum hardness principle, chemical hardness is a measure of the chemical stability and hard molecules are more stable compared to soft ones [62]. Some researchers explained that hard molecules exhibit resistance to charge transport. The hardest one of studied molecules is BIPY molecule and this molecule with a high electron reorganization energy can be used as an electron blocking materials because of its hard property. The inverse relation between hardness and polarizability was reported by Ghanty and Ghosh with a note of which softness (multiplicative inverse of the hardness) is proportional to the cube root of the polarizability (α) [63]. Considering the inverse relation between hardness and polarizability, Chattaraj proposed the minimum polarizability principle and reported that in stable state, polarizability is minimized [64]. In Table 2, calculated quantum chemical parameters for the studied chemical systems are given. It can be seen from the data that the hardness molecule, BIPY has the lowest value of polarizability. Namely, when hardness is maximized, polarizability is minimized. Another well-known electronic structure principle is minimum electrophilicity principle but von Szentpály et al. [65] made some critical comments regarding the validity of minimum electrophilicity principle states that stable states correspond to the minimum value of the electrophilicity index.

### 3.3. Effective transfer integrals and charge transfer rates

To calculate the effective transfer integrals for the studied molecules, dimeric structures of molecules are considered. Therefore, the effective transfer integrals can be calculated among the two compounds which are called dimer. For that reason, the dimer structures of the investigated molecules are determined at B3LYP/TZP level and given in Figure. Additionally, Figure shows the N-N distance among dimer structures. Using dimer structures in Figure, the effective transfer integrals of the aforementioned molecules are computed and tabulated in Table 3. It is apparent from the result given in Table 3 that BISTERPY molecule has the highest absolute transfer integrals which are electron and hole transfer integrals. Therefore, it can be said that the good electron and hole transfer material is BISTERPY. In other words, it should be noted that BISTERPY molecule is a candidate as ambipolar material.

With the help of Marcus-Hush equation appearing by Eq. 1, both electron and hole charge transfer rates of the molecules can be calculated. The charge transfer rates of the molecules are obtained using Marcus-Hush formula and are presented in Table 3. From Table 2, it is seen that the electron and hole charge transfer rates of BISTERPY compound are calculated as 4.93 × 10^13^ and 2.77 × 10^13^ s^−1^, respectively. Considering these values, one can say that the charge transfer rates of BISTERPY compound have the highest values among the investigated molecules. In order to conclude, it can be stated that BISTERPY is both electron and hole transfer materials which is called ambipolar because of its high charge transfer rates.

## 4. Conclusion

OLED behaviors of some aromatic nitrogen-containing ligands are determined using quantum chemical descriptors. Based on concept of reorganization energy, it can be stated that BISTERPY molecule is good ETL and HTL materials. Addition to this, one can say that DABP and AZPY compounds are candidates as EBL and HBL materials, respectively. Similarly, using ionization potential and electron affinity values, it can be predicted that DABP and AZPY molecules can be used as HIL and EIL materials. Finally, with the help of effective transfer integrals and charge transfer rates, one can say that BISTERPY compound is an excellent candidate as charge transfer materials. The investigated molecules can be also suggested for other optoelectronic devices. (1)

## Figures and Tables

**Figure:**
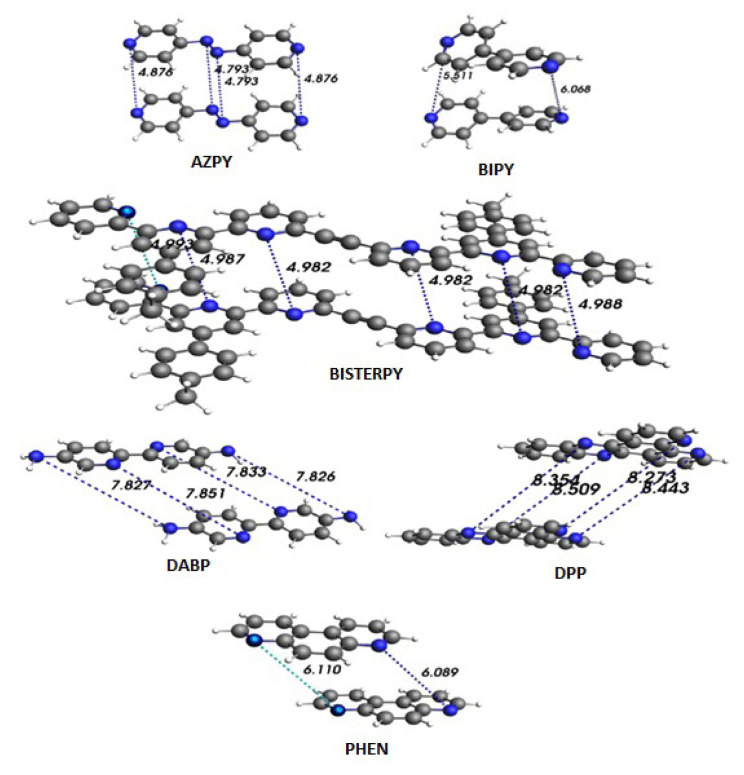
The dimer structures and the nitrogen-nitrogen distances of the investigated compounds at B3LYP/TZP level.

**Scheme:**
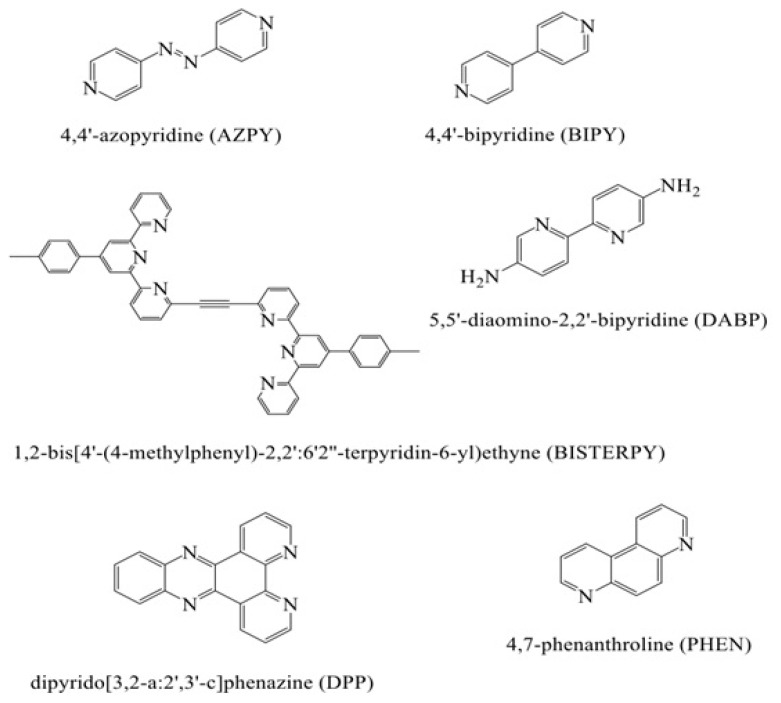
The structure of the studied molecules.

**Table 1:** The obtained reorganization energy, adiabatic/vertical ionization potential, adiabatic/vertical electron affinity, and chemical hardness values (all in eV) of the mentioned compounds at different functional with 6-31G(d) basis set in the vacuo.

Compound	λe	λh	IPa	IPv	EAa	EAv
B3LYP/6–31G						
Alq3	0.26	0.23	6.23	6.32	0.44	0.54
TPD	0.46	0.28	5.58	5.71	−0.37	−0.12
AZPY	0.56	1.51	8.11	8.78	1.55	1.27
BIPY	0.62	0.53	8.66	8.92	0.23	−0.12
BISTERPY	0.14	0.12	6.82	6.87	0.66	0.59
DABP	0.72	0.62	6.28	6.65	−0.70	−1.04
DPP	0.21	0.37	7.71	8.01	1.07	0.97
PHEN	0.36	0.49	8.02	8.41	0.04	−0.14
B3LYP-D3/6-31G						
Alq3	0.28	0.23	6.24	6.32	0.45	0.58
TPD	0.53	0.26	5.58	5.70	−0.39	−0.12
AZPY	0.56	1.58	8.12	8.78	1.55	1.27
BIPY	0.61	0.52	8.66	8.92	0.23	−0.12
BISTERPY	0.14	0.13	6.82	6.87	0.65	0.57
DABP	0.72	0.62	6.28	6.65	−0.70	−1.04
DPP	0.20	0.12	7.70	7.77	1.07	0.97
PHEN	0.36	0.21	8.02	8.12	0.04	−0.14
CAM-B3LYP/6-31G						
Alq3	0.91	0.76	6.66	7.03	−0.12	0.44
TPD	0.73	0.60	5.89	6.17	−0.74	−0.30
AZPY	0.71	1.12	8.98	9.60	1.50	1.14
BIPY	0.73	1.07	8.95	9.54	0.15	−0.27
BISTERPY	0.71	0.40	7.75	7.92	0.25	−0.20
DABP	0.85	2.43	6.84	8.62	−0.78	−1.17
DPP	0.28	0.59	8.36	8.63	0.99	0.85
PHEN	0.45	0.51	9.12	9.37	−0.06	−0.28
ωB97X-D/6-31G(d)						
Alq3	0.69	0.78	6.38	6.77	0.09	0.44
TPD	0.89	0.67	5.99	6.31	−0.78	−0.29
AZPY	0.72	1.76	8.34	9.11	1.53	1.17
BIPY	0.76	1.16	8.98	9.66	0.15	−0.27
BISTERPY	0.54	0.48	7.67	7.92	0.26	−0.03
DABP	0.86	2.41	6.89	8.65	−0.77	−1.15
DPP	0.28	0.62	8.37	8.66	1.03	0.89
PHEN	0.45	0.53	9.18	9.45	−0.05	−0.26

**Table 2:** The calculated quantum chemical descriptors (in eV) for studied molecules at B3LYP/6-31G(d) level.

Compound	η	χ	ω	ω^+^	ω^−^	α (a.u)
Alq3	1.64	3.37	3.46	1.98	5.35	327.27
TPD	1.95	2.72	1.90	0.78	3.51	475.23
AZPY	1.92	4.91	6.29	4.07	8.99	142.27
BIPY	2.71	4.37	3.52	1.68	6.05	109.58
BISTERPY	2.12	3.68	3.20	1.63	5.31	605.99
DABP	2.20	2.81	1.79	0.66	3.47	144.50
DPP	1.95	4.40	4.96	3.00	7.40	234.52
PHEN	2.41	3.98	3.28	1.59	5.57	136.50

**Table 3:** The determined electron and hole transfer integrals (in eV) and the electron and hole charge transfer rates (s^−1^) of the investigated molecules at hybrid B3LYP method in the gas phase.

Molecule	V_e_	V_h_	We	Wh
AZPY	0.02782	−0.02648	7.71 × 10^10^	3.95 × 10^6^
BIPY	0.00529	0.00132	1.48 × 10^9^	2.37 × 10^8^
BISTERPY	0.06551	0.04282	4.93 × 10^13^	2.77 × 10^13^
DABP	0.00489	−0.02043	4.11 × 10^8^	2.18 × 10^10^
DPP	−0.00430	−0.00211	8.78 × 10^10^	3.32 × 10^9^
PHEN	0.00453	0.00552	1.81 × 10^10^	5.95 × 10^9^
